# Supplementary material for: Complexome profiling of the Chlamydomonas psb28 mutant reveals TEF5 as an early PSII assembly factor
Source: Plant Cell. 2025 Mar 17;37(6):koaf055. doi: 10.1093/plcell/koaf055 (PMC12164590; doi:10.1093/plcell/koaf055)
Supplement: koaf055_Supplementary_Data [file koaf055_supplementary_data.zip › psb28_interactive.zip!/plots/intensity/qualityPlots/peptides/rps2_Cre-1.g2717005.t1.p1.html]

### Cre-1.g2717005.t1.p1

Trivial name: rps2  
SeqCoverage [median]: 8.20  
Mass: 24.37 kDa  
  
 

| Sequence | MissedCleavage | Mass [Da] | wtIntensity | muIntensity | Score |
| --- | --- | --- | --- | --- | --- |
| QTLQTER | 0 | 874.45 | 0.00 | 377.44 | 111.04 |
| YLGGISNMTK | 0 | 1082.54 | 0.00 | 378.38 | 71.50 |
|  |  |  | 0.0 | 755.8 |  |
